# Rapid Detection of Chicken Infectious Anemia Virus Using a One-Tube RPA-CRISPR/Cas12a System

**DOI:** 10.3390/vetsci13060529

**Published:** 2026-05-29

**Authors:** Lei Ma, Mengjie Zhu, Yajie Tang, Xueping Wang, Xiaojun Zhang

**Affiliations:** 1 School of Biotechnology and Food Engineering, Anyang Institute of Technology, Anyang 455000, China; 2 College of Animal Science and Technology, Tarim University, Alar 843320, China

**Keywords:** chicken infectious anemia virus, Cas12a, Recombinase polymerase amplification, detection

## Abstract

Chicken infectious anemia virus (CIAV) is a major immunosuppressive pathogen that causes significant economic losses to the global poultry industry. Conventional detection methods for CIAV are limited by poor timeliness, high equipment requirements, and insufficient sensitivity. To address these challenges, this study developed a novel one-tube integrated RPA-CRISPR/Cas12a assay targeting the highly conserved VP3 gene of CIAV for rapid and accurate detection. The performance of the assay was comprehensively evaluated in terms of sensitivity, specificity, and repeatability. Its clinical utility was assessed by testing 80 clinical suspected samples, with quantitative real-time PCR (qPCR) serving as the reference method. The results showed that the limit of detection (LoD) of the developed method was 10 copies/reaction, comparable to that of qPCR. No cross-reactivity with common avian pathogens was observed. The intra- and inter-assay coefficients of variation (CV%) for the time to threshold (Tt) were both below 10%. In clinical sample detection, the assay achieved a total coincidence rate of 97.5%, with a sensitivity of 100% and specificity of 96% relative to qPCR. In conclusion, the RPA-CRISPR/Cas12a assay developed in this study offers rapid detection, high sensitivity and specificity, operational simplicity, low equipment dependency, and excellent repeatability. It provides a practical tool for early and rapid diagnosis of CIAV, clinical sample screening in grassroots veterinary laboratories, and on-site epidemiological surveillance in poultry farms, holding significant potential for the precise prevention, control, and eradication of CIAV in the poultry industry.

## Simple Summary

Chicken infectious anemia virus (CIAV) causes severe immunosuppression and substantial economic losses in the poultry industry. Conventional detection methods are limited by slow speed, high equipment requirements, or insufficient sensitivity. To address these issues, we developed a one-tube RPA-CRISPR/Cas12a assay targeting the conserved VP3 gene of CIAV. The assay enables rapid detection at a constant temperature of 39 °C within 40 min, with a limit of detection of 10 copies per reaction, comparable to quantitative real-time PCR (qPCR). It shows no cross-reactivity with other common avian pathogens and exhibits excellent repeatability, with coefficients of variation below 10%. Testing 80 clinical samples yielded 100% sensitivity and 96% specificity relative to qPCR. This one-tube assay is simple, fast and sensitive, making it a practical tool for early diagnosis, field surveillance, and clinical screening of CIAV in grassroots veterinary laboratories and poultry farms.

## 1. Introduction

Chicken Infectious Anemia (CIA), also referred to as Blue Wing Disease, or Hemorrhagic Syndrome, is primarily induced by infection with Chicken infectious anemia virus (CIAV) [1]. Belonging to the family Circoviridae and the genus Gyrovirus, CIAV is a single-stranded circular DNA virus [2]. This virus possesses icosahedral symmetry and exists as a non-enveloped viral particle, presenting a spherical or hexagonal morphology under electron microscopy. CIAV is capable of causing a variety of immunosuppressive disorders, including atrophy of immune organs and tissues, subcutaneous and muscular hemorrhage, as well as aplastic anemia of the hematopoietic system.

CIA mainly occurs in chicks aged 2–3 weeks. Pathological changes following CIAV infection are mainly characterized by bone marrow lipomatosis, thymic atrophy, and impaired blood coagulation. Affected chickens typically present with dark red bone marrow accompanied by thymic atrophy or complete regression [3,4]. Histologically characteristic changes include a severe reduction in bone marrow hematopoietic cells, which are replaced by adipose tissue or proliferating stromal cells, ultimately resulting in regenerative anemia. The thymic cortex exhibits significant lymphocyte depletion, cellular edema and degeneration, generalized lymphoid tissue atrophy, as well as severe lymphocyte deficiency in organs such as the bursa of Fabricius, spleen, and cecal tonsils [5].

CIAV has been detected in numerous countries worldwide, including the United Kingdom, Germany, Australia, the United States and Nigeria [6,7,8,9,10]. Currently, CIAV is widely prevalent in China, with a high infection rate in poultry flocks, and the natural infection rate can reach 70~100%. CIAV infection has been documented in almost all provinces across China [3,11,12,13,14]. CIAV can infect young chickens through both vertical and horizontal transmission routes. Infected flocks are in a state of immunosuppression, which increases their susceptibility to other pathogens such as Aspergillus fumigatus. Meanwhile, CIAV-induced immunosuppression impairs the host immune responsiveness, weakening the protective effect of vaccines against Newcastle disease virus (NDV) and other pathogens, thereby leading to increased mortality during concurrent or secondary infections [15,16,17,18,19]. These consequences pose significant challenges to the broiler industry and result in substantial economic losses. Timely diagnosis and culling of infected chickens, followed by harmless disposal (e.g., deep burial, incineration), are crucial for preventing viral spread and controlling the disease. Therefore, establishing a rapid and sensitive diagnostic method is particularly important for the prevention and control of CIAV.

Diagnostic methods for CIAV mainly include viral isolation, identification, PCR-based diagnostic techniques, nested PCR, indirect immunofluorescence assay, enzyme-linked immunosorbent assay (ELISA), viral neutralization test and so on [20,21,22,23,24]. Although each method has its own advantages, they all have limitations in terms of sensitivity, specificity, and timeliness. Cultivation using cells or chicken embryos shows relatively low in vitro replication capacity and requires prolonged detection time. Quantitative real-time PCR (qPCR) exhibits high specificity but necessitates professional expertise and expensive equipment.

In recent years, isothermal amplification technologies represented by recombinase polymerase amplification (RPA) and specific nucleic acid recognition technologies based on the CRISPR-Cas system have brought revolutionary breakthroughs to molecular diagnostics [25,26,27]. RPA technology enables rapid and efficient amplification of target nucleic acids under constant temperature conditions (37–42 °C), eliminating the dependence on precision instruments. The CRISPR-Cas12 protein, guided by guide RNA (gRNA), can specifically recognize double-stranded DNA targets and subsequently exhibit non-specific “trans-cleavage” activity, which efficiently cleaves reporter molecules and converts nucleic acid recognition signals into easily readable fluorescent or lateral flow chromatography signals. Based on this, the present study aims to innovatively develop a novel RPA-CRISPR/Cas12a combined detection method for the high-sensitivity and high-specificity detection of CIAV. We designed specific RPA primers and Cas12a-crRNA targeting the conserved gene sequences of CIAV. By optimizing the reaction system and conditions, we achieved a one-tube, constant-temperature, and rapid detection process (typically completed within 1 h), with results visualized through fluorescence reading. This method is expected to provide a simple-to-operate, cost-controllable, and reliable real-time detection tool for CIAV, thereby offering strong technical support for the early warning and integrated prevention and control of CIA.

## 2. Materials and Methods

### 2.1. Target Gene Selection and Sequence Analysis

To determine the conserved gene region, the full-length gene sequences of eight CIAV strains from different regions worldwide were downloaded from the NCBI GenBank database, and multiple sequence alignment was performed using DNAstar software 7.0 to exclude regions with high mutation rates. Based on the alignment results, a gene fragment with 99% sequence consistency among different CIAV strains was identified.

### 2.2. Construction and Preparation of Standard Plasmids

The nucleic acid of a CIAV-positive sample was used as the template, and traditional PCR primers (5′-ACGGCGGACAACCGGCCGCT-3′, 5′-ACTCACGGCGATGGGGCCT-3′) designed for the conserved region of the VP3 gene were adopted for amplification. The PCR product was first verified by 1.0% agarose gel electrophoresis to confirm the target band size, and then the target fragment was recovered and purified by gel extraction. The purified PCR product was cloned into the pMD18-T vector by T-A cloning technology for ligation reaction, and the ligation product was transformed into DH5α competent cells and cultured overnight at 37 °C. Single colonies were picked for PCR verification to screen positive clones. The positive recombinant plasmid (named pMD19T-VP3) was extracted using a plasmid extraction kit. A NanoDrop 2000 spectrophotometer (Thermo Fisher Scientific, Wilmington, DE, USA) was used to determine the plasmid concentration (ng/μL). The copy number of the qualified recombinant plasmid was calculated. The recombinant plasmids were subjected to 10-fold serial gradient dilution with sterile TE buffer to obtain concentrations ranging from 10^6^ to 10^0^ copies/μL. The diluted plasmids were aliquoted and stored at −80 °C as standard products for subsequent experiments, and repeated freeze–thaw was avoided to ensure the stability of the standard products.

### 2.3. Design of RPA Primers and CrRNA

RPA primers were designed for the conserved region of the CIAV VP3 gene in accordance with the design principles of RPA technology [28,29,30,31]. Based on the above principles, a total of three primer pairs were designed. All candidates were screened by RPA followed by nucleic acid agarose gel electrophoresis. The primer pair that yielded clear target bands without non-specific amplification was finally selected and listed in Table 1. The detailed RPA primer sequences and electrophoresis validation data have been supplemented and placed in the Supplementary Materials.

CrRNA was designed for the CIAV RPA amplicon to match the recognition characteristics of the Cas12a protein, and the design followed strict principles: the CrRNA sequence must contain the TTTV (V=A/C/G) sequence specifically recognized by Cas12a, and the PAM site selected in this study was TTTC, which is located within the RPA amplicon; a 20 nt specific sequence was selected upstream of the 5′ end of the PAM sequence, which was completely complementary to the CIAV VP3 gene with no base mismatches; the complete CrRNA sequence was composed of a T7 promoter sequence, a 20 nt target sequence and a Cas12a scaffold sequence (UAAUUUCUACUAAGUGUAGA), in which the scaffold sequence could ensure the effective binding of CrRNA and Cas12a protein. Based on the above design principles, three specific CrRNAs were designed in this study. (Table 1). The CrRNA DNA template containing the T7 promoter was synthesized by Sangon Biotech (Shanghai) Co., Ltd. (Shanghai, China) and cloned into the pGEX-T transcription vector. High-purity CrRNA was obtained by in vitro transcription using a T7 RNA polymerase in vitro transcription kit, and the transcribed product was purified. The concentration of the purified CrRNA was determined, and a 100 μM stock solution was prepared by dilution, then aliquoted and stored at −80 °C. The stock solution was diluted to the required working concentration before use in subsequent experiments. The sequences of the RPA primers and CrRNAs were listed in Table 1.

### 2.4. Evaluation of the CrRNA Activity

To evaluate the activity of CrRNA, we performed CRISPR/Cas12a detection and analysis. The reaction protocol contained 0.5 μL of Cas12a protein (New England Biolabs, Ipswich, MA, USA), 1 μL of CrRNA, (2 μM), 2 μL of 10× NE Buffer 2.0 (New England Biolabs, USA), 0.5 μL of single-stranded DNA (ssDNA) probe (5′-FAM-TTATT-BHQ1-3′) as the reporter molecule, and 2 μL of the pMD-VP3 plasmid. The reaction tube was incubated at 39 °C for 20 min using a fluorescence detector (Deao, Guangzhou, China) for fluorescence signal detection. In addition, negative controls were set up, including no-template control (NTC) and the CrRNA-free control (CrRNA replaced with distilled water). The fluorescence intensity was recorded and used to evaluate the cleavage efficiency of CrRNA.

### 2.5. Establishment of One-Tube RPA-CRISPR/Cas12a Detection System

To achieve closed-tube detection and minimize the risk of aerosol contamination, a separate one-tube assembly method of “bottom-RPA reaction system + tube lid-CRISPR detection system” was adopted in this study, and all experimental operations were performed on ice to preserve the activity of reagents. The RPA premix was prepared at the bottom of a 0.2 mL PCR tube with a total volume of 50 μL, including 29.5 μL Rehydration Buffer, 2.4 μL RPA-F (10 μM), 2.4 μL RPA-R (10 μM), 1 μL template nucleic acid (standard product or clinical sample), and 11.7 μL nuclease-free water. Finally, 2.5 μL of 280 mM magnesium acetate (MgOAc) was added to initiate the RPA reaction (the lyophilized enzyme powder of the RPA kit was preset at the bottom of the PCR tube).

The CRISPR detection solution, with a total volume of 5.5 μL, was spotted on the inner wall of the PCR tube lid, consisting of 1 μL Cas12a protein, 1 μL crRNA-CIAV, 1 μL ssDNA fluorescent reporter probe (5′-FAM-TTATT-BHQ1-3′, 10 μM), 0.25 μL RNase inhibitor (40 U/μL), and 2 μL 10× NEBuffer 2.0. The tube lid was carefully closed to prevent the CRISPR detection solution on the lid from dripping into the RPA premix at the bottom of the tube. Immediately afterward, the tube was placed in a preheated metal bath at 39 °C and incubated for 20 min to complete the RPA reaction. After the RPA reaction, the PCR tube was briefly centrifuged to completely transfer the CRISPR detection solution on the tube lid to the bottom of the tube. The tube was then transferred to a fluorescence detector (Deao, Guangzhou, China) maintained at 39 °C for subsequent fluorescence detection. Fluorescence signals were collected every 30 s for a total of 40 cycles, and the fluorescence kinetic curve was recorded.

The criteria for judging the detection results were as follows: a typical “S”-shaped exponential increase in the fluorescence curve, with the fluorescence value exceeding the mean value of the negative control plus 3 times the standard deviation, was judged as positive; no obvious increase in the fluorescence curve, with the fluorescence signal consistent with that of the negative control, was judged as negative.

### 2.6. Optimization of CrRNA and Cas12a Protein Concentrations

To obtain the optimal detection performance of the system, the working concentrations of CrRNA and Cas12a protein were optimized, with the highest fluorescence signal intensity and the shortest time to threshold (Tt) set as the optimization criteria. For the optimization of CrRNA concentration, the Cas12a protein concentration was fixed at 1.0 μM, and four CrRNA concentration gradients (0.25, 0.5, 1.0, and 1.5 μM) were tested. Then, the CrRNA concentration was fixed at the optimal concentration, and four Cas12a protein concentration gradients (0.25, 0.5, 1.0, and 1.5 μM) were tested.

### 2.7. Sensitivity

To evaluate the sensitivity of the established detection method, the pMD19T-VP3 standard product with serial gradient dilutions of 10^6^–10^0^ copies/μL was used as the template, and each concentration was tested in 3 independent replicates. The limit of detection (LoD) of the method was defined as the lowest template concentration that could stably produce positive fluorescence signals with a 100% positive rate in replicate tests.

### 2.8. Specificity

For specificity validation, the established method was used to detect the nucleic acids of common avian pathogens (including Avian Leukosis Virus ALV-J, Marek’s Disease Virus MDV, Infectious Bursal Disease Virus IBDV, Fowl Adenovirus Serotype 4 FAdV-4, Newcastle Disease Virus NDV, Infectious laryngotracheitis virus ILTV, Avian Influenza Virus H9N2 and H3N8), using genomic DNA of the healthy chicken and distilled water as the negative control. All the above pathogens were described in our previous report [30]. The CIAV-positive sample used in the specificity test was confirmed by conventional PCR established in our laboratory. Meanwhile, other common avian viral pathogens were simultaneously detected in this sample, and all presented negative results. Therefore, this sample was used as the CIAV positive control in this study. The method was considered to have good specificity only when the CIAV-positive sample was detected as positive, and all other non-target pathogen nucleic acids, chicken genomic DNA and negative control were detected as negative.

### 2.9. Repeatability

The repeatability of the developed method was assessed in terms of intra-assay and inter-assay variation, using endpoint fluorescence values at the end of the reaction as the evaluation index. The mean value, standard deviation (SD), and coefficient of variation (CV%) were calculated based on these endpoint fluorescence data. A CV% below 10% was defined as acceptable good repeatability for the method. For intra-assay repeatability, the same operator used the same reagent batch and the same instrument to perform 3 parallel tests on the standard products with three concentrations of 10^6^, 10^4^ and 10^2^ copies/μL. For inter-assay repeatability, different operators used different reagent batches to perform 3 replicate tests on the above three concentrations of standard products on different experimental dates.

### 2.10. Clinical Samples

To evaluate the clinical application value of the established method, a total of 80 clinical samples were collected from several farms in Henan Province during 2020–2025. Specifically, one thymus was harvested from each chicken. Notably, these samples were originally obtained for routine diagnostic testing rather than specifically for the present study; thus, relevant animal ethical review and approval were not applicable. The nucleic acids of the samples were extracted from the samples. Each 1 g clinical sample was homogenized, mixed with 200 µL QIAGEN ATL lysis buffer, and incubated at 70 °C for 20 min [32]. Following high-speed centrifugation, the supernatant containing purified nucleic acids was harvested and used for subsequent detection assays. OD260/280 ratios of the extracted nucleic acids were determined by NanoDrop (ThermoFisher, Waltham, MA, USA).

The CIAV-specific quantitative real-time PCR (qPCR) was used as the gold standard. The detailed information of the qPCR was described previously [33]. Diagnostic indicators including sensitivity, specificity, positive predictive value (PPV) and negative predictive value (NPV) were calculated based on the 2 × 2 contingency table. The 95% confidence intervals (95% CIs) of all indicators were estimated using the Clopper–Pearson exact binomial method. Linear regression analysis was performed using GraphPad Prism software 7.0 to explore the association between the threshold time (TT) obtained from the RPA-CRISPR/Cas12a assay and the cycle threshold (Ct) values determined by qPCR. The coefficient of determination (R^2^) was calculated to evaluate the linear association between the two detection methods.

## 3. Results

### 3.1. Determination of Highly Conservative Sequence Regions

**Figure 1:**
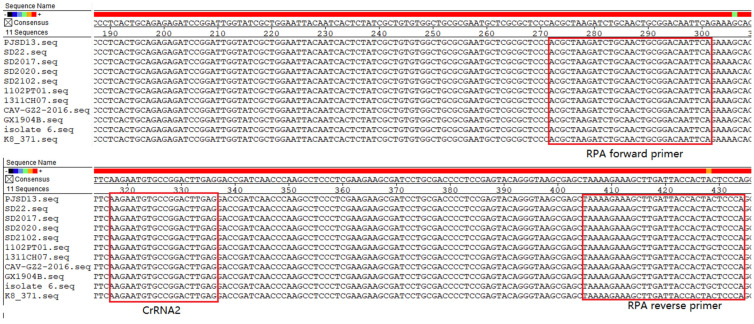
Sequence alignment of the target gene. RPA primers and CrRNA are indicated in red boxes.

Based on the high conservation of the CIAV VP3 gene, a 165bp fragment at positions 801–965 of the gene was selected as the detection target region in this study. Sequence alignment results showed that this fragment had 99% sequence consistency among eight CIAV strains (Figure 1), and had no sequence homology with the chicken genome and other common avian pathogen genomes, which laid a solid foundation for the specific detection of CIAV.

As illustrated in Figure 1, the sequence alignment results of the primer-binding regions clearly demonstrate that both the upstream and downstream primer sequences exhibit extremely high conservation among all the CIAV strains from different geographical regions included in the analysis. No base deletions and insertions were observed in the primer-binding sites across these strains. There is a single nucleotide discrepancy (G vs. C) in the reverse primer. A degenerate base Y was used to substitute this position to ensure primer efficiency.

### 3.2. Screening of CrRNA

**Figure 2:**
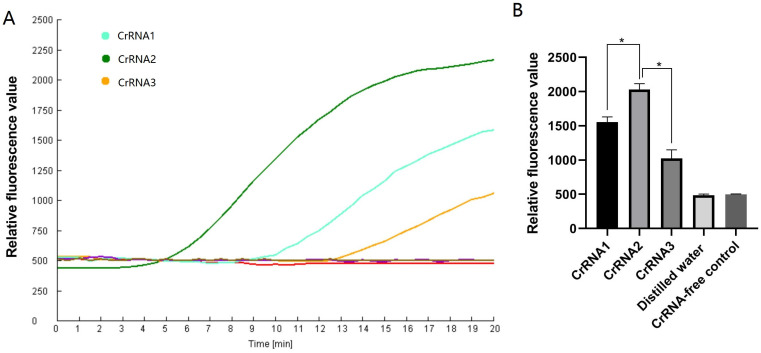
Screening of the optimal crRNA. The efficiency of different crRNAs was evaluated using the Cas12a-based detection assay. (**A**) Fluorescence curves of the detection signals; (**B**) Bar graph showing quantitative analysis of three independent replicates. * indicates a significant difference at *p* < 0.05.

Recombinant DNA plasmids (pMD-VP3) were used as detection templates to assess the cleavage activity of three candidate CrRNAs. As illustrated in Figure 2, real-time fluorescence curves are displayed in Figure 2A, whereas Figure 2B presents a bar chart summarizing three independent replicates with corresponding statistical significance analysis. The other independent tests were presented in the Supplementary Materials.

All three CrRNAs produced clear and discernible fluorescence curves. Notably, CrRNA2 exhibited a significantly higher fluorescence signal intensity compared with both CrRNA1 and CrRNA3. Accordingly, CrRNA2 was ultimately selected for subsequent experiments.

### 3.3. Optimization of CrRNA and Cas12a Protein Concentrations

**Figure 3:**
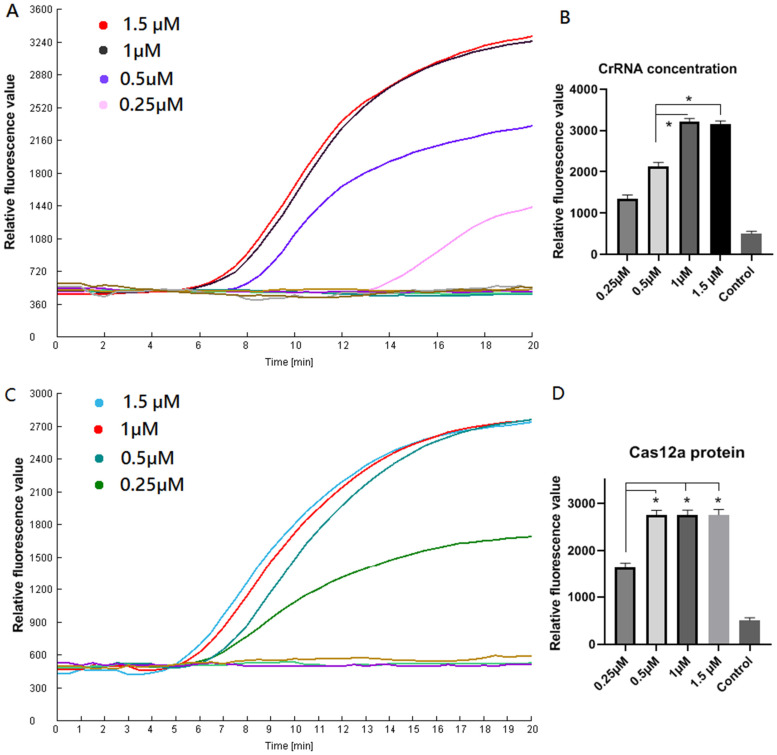
Optimization of CrRNA and Cas12a concentrations for the one-tube RPACRISPR/Cas12a assay. (**A**) CrRNA was tested at final concentrations of 0.25, 0.5, 1, and 1.5 µM. (**B**) All reactions were performed in triplicate. (**C**) Cas12a was evaluated at final concentrations of 0.25, 0.5, 1, and 1.5 µM. (**D**) All reactions were performed in triplicate. * indicates a significant difference at *p* < 0.05.

The results showed that when the CrRNA concentration increased from 0.25 μM to 1.0 μM, the maximum fluorescence intensity increased significantly, while there was no significant change in fluorescence intensity when the CrRNA concentration increased from 1.0 μM to 1.5 μM, so 1.0 μM was determined as the optimal CrRNA concentration (Figure 3A,B).

To optimize the Cas12a protein concentration, the CrRNA concentration was fixed at 1.0 μM, and four gradient levels of Cas12a (0.25, 0.5, 1.0, and 1.5 μM) were evaluated. Although comparable maximum fluorescence intensities were observed at 0.5, 1.0, and 1.5 μM; the Ct values at 1.0 and 1.5 μM were notably lower than that at 0.5 μM. On these grounds, 1.0 μM was chosen as the optimal concentration of Cas12a protein (Figure 3C,D). Accordingly, the final optimized one-tube RPA-CRISPR/Cas12a reaction system was determined to comprise 1.0 μM CrRNA and 0.5 μM Cas12a protein.

### 3.4. Sensitivity

**Figure 4:**
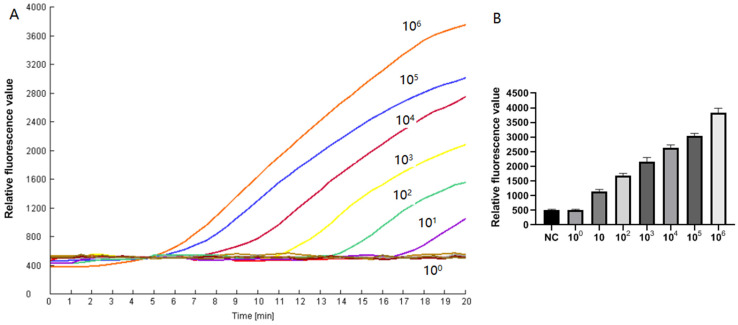
Analytical sensitivity of the RPA-CRISPR/Cas12a assay. (**A**) The limit of detection was determined using ten-fold serially diluted standards as templates. (**B**) All assays were performed in triplicate.

The sensitivity detection results showed that when the pMD19T-VP3 standard product with serial gradient dilutions of 10^6^–10^0^ copies/μL was used as the template, the samples with concentrations from 10^6^ to 10^0^ copies/μL all produced typical fluorescence growth curves, and the fluorescence signal initiation time was gradually delayed with the decrease in the template concentration. The negative control had a stable background throughout the reaction process with no obvious fluorescence signal increase. This method could stably and repeatedly detect the CIAV genome with a concentration as low as 10 copies/μL. However, the template with a concentration of 1 copy/μL tested negative (Figure 4A,B). Therefore, the limit of detection (LoD) of this method was determined as 10 copies/μL, which was comparable to the gold standard qPCR in terms of sensitivity. We have performed two independent experiments, with 10 replicates tested in each experiment at the concentration of 10 copies/reaction. The results confirmed a 100% positive detection rate, and the relevant data are provided in the Supplementary Materials.

### 3.5. Specificity

**Figure 5:**
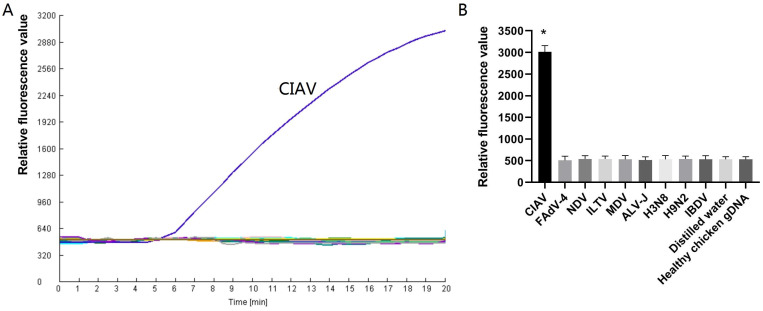
Analytical specificity of the fluorescence-based RPA-CRISPR/Cas12a assay. (**A**) Detection results for chicken infectious anemia virus (CIAV) and other common avian pathogens including ALV-J, MDV, IBDV, FAdV-4, ILTV, NDV, AIV-H9N2 and H3N8; (**B**) Quantitative analysis of results from three technical replicates. * represents a significant difference at *p* < 0.05.

The specificity detection results showed that only the CIAV-positive sample produced a significant increase in fluorescence signal within 5 min. The detection results of other common avian pathogens (ALV-J, MDV, IBDV, FAdV-4, ILTV, NDV and AIV-H9N2) and chicken genomic DNA were completely consistent with the negative control, with no fluorescence signal increase during the whole reaction process and no non-specific cross-reactivity (Figure 5A,B). This indicated that the established method had high species specificity for CIAV, and could effectively distinguish CIAV from other avian pathogens.

### 3.6. Repeatability Test

The results of the repeatability test showed that for the CIAV standard products with three concentrations of high (10^6^ copies/μL), medium (10^4^ copies/μL) and low (10^2^ copies/μL), the intra-assay and inter-assay coefficient of variation (CV%) of the fluorescence value were all below 10% (Table 2). The above results indicated that the established RPA-CRISPR/Cas12a detection method had excellent repeatability and intra-laboratory stability, and the detection results were reliable with little influence from operators, experimental dates and reagent batches.

### 3.7. The Result of the Clinical Detection Performance

To verify the clinical detection performance of the method, 80 clinical suspected CIAV-infected chicken tissue samples were collected for parallel detection by the established method and the qPCR method. In this study, we further evaluated the nucleic acid purity of all clinical samples, and the OD260/280 ratios of all samples were within the acceptable range of 1.8–2.0.

Of these samples, 32 CIAV-positive samples and 48 CIAV-negative samples were determined by RPA-CRISPR/Cas12a detection method. Meanwhile, 30 CIAV-positive samples and 50 CIAV-negative samples were determined by qPCR. All the CIAV-positive samples determined by qPCR tested positive by RPA-CRISPR/Cas12a method. Of the 50 CIAV-negative samples by qPCR, 2 samples tested positive by the RPA-CRISPR/Cas12a method (Table 3). The raw data of the detection results is provided in Supplementary Materials.

Based on the qPCR-verified 80 clinical samples (30 positive and 50 negative), the RPA-CRISPR/Cas12a assay produced 30 true positive (TP), 2 false positive (FP), 0 false negative (FN), and 48 true negative (TN). Diagnostic parameters were calculated as follows: Sensitivity = TP/(TP + FN) = 30/(30 + 0) = 100.00% (95% CI: 88.43–100.00%); Specificity = TN/(TN + FP) = 48/(48 + 2) = 96.00% (95% CI: 86.29–99.32%); PPV = TP/(TP + FP) = 30/(30 + 2) = 93.75% (95% CI: 79.01–98.91%); and NPV = TN/(TN + FN) = 48/(48 + 0) = 100.00% (95% CI: 92.60–100.00%). Cohen’s Kappa coefficient was determined to be 0.947, indicating an excellent detection consistency between the RPA-CRISPR/Cas12a assay and the qPCR gold standard.

**Figure 6:**
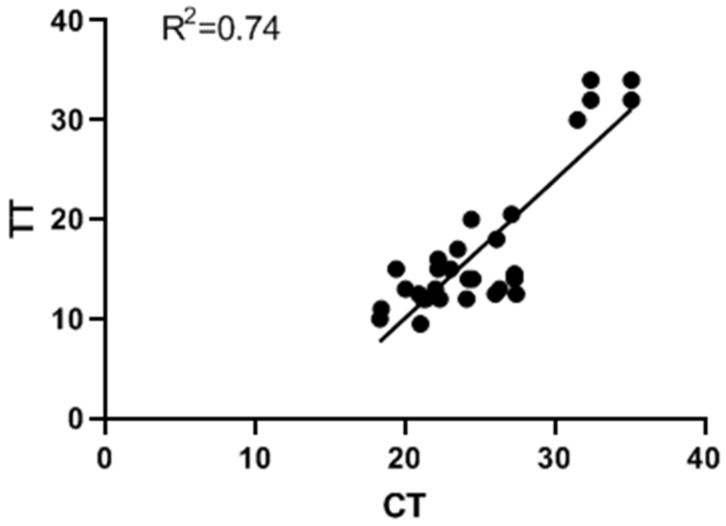
Linear regression analysis was performed to compare the threshold time (TT) values from the RPA-CRISPR/Cas12a assay and cycle threshold (CT) values from qPCR.

Linear regression analysis was performed to investigate the association between the threshold time (TT) values from the RPA-CRISPR/Cas12a assay and cycle threshold (Ct) values from qPCR, rather than direct numerical comparison. The coefficient of determination (R^2^) was calculated to be 0.74 (Figure 6). This moderate association indicated that the established RPA-CRISPR/Cas12a method was suitable for qualitative detection of CIAV, but was not sufficiently reliable for precise quantitative analysis.

**Table 3:** Comparison of the detection results between RPA-CRISPR/Cas12a and qPCR.

		qPCR		
		Positive	Negative	Total
RPA-CRISPR/Cas12a	Positive	TP = 30	FP = 2	32
	Negative	FN = 0	TN = 48	48
	Total	30	50	80

## 4. Discussion

In this study, with the highly conserved VP3 gene of CIAV as the target, a novel integrated one-tube RPA-CRISPR/Cas12a rapid detection method for CIAV was successfully constructed and established. The optimal reaction condition of this method was determined as CrRNA 1.0 μM combined with Cas12a protein 1.0 μM, and the whole detection process could be completed by constant temperature incubation at 39 °C for 40 min. The method adopted a closed-tube detection mode with simple operation steps, which could significantly reduce the risk of aerosol contamination in the detection process. The sensitivity of the established method reached 10 copies/reaction, which was comparable to qPCR. Meanwhile, the method had high species specificity for CIAV, with no cross-reactivity with other common avian pathogens, and excellent repeatability and stability. Repeatability analysis showed that the intra-assay and inter-assay CV% ranged from 5.2% to 8.0% and 5.7% to 8.4%, respectively. The relatively low CV values indicated excellent repeatability, stability and reliability of the established method. The detection performance in clinical samples further verified the reliability of the established RPA-CRISPR/Cas12a assay. Using qPCR as the gold standard, this method presented 100.00% sensitivity, 96.00% specificity, 93.75% PPV and 100.00% NPV in 80 clinical samples. In addition, the method did not require expensive precision instruments, and could be matched with portable constant temperature incubators and fluorescence detectors to realize on-site rapid detection.

The VP capsid protein genes of CIAV are characterized by high genetic conservation and stable genomic traits, which serve as a key molecular basis for the development of reliable detection assays. Among existing diagnostic strategies, the VP1 gene has been widely exploited as the target for nucleic acid tests [34,35,36,37]. However, through systematic sequence alignment and comparative analysis in this study, we verified that the VP3 gene of CIAV also harbors exceptional conservation across different isolates. Multiple sequence alignment results confirmed that the target region of the VP3 gene shared a 99% sequence identity across all tested CIAV strains, with no detectable homology to the chicken genome or major avian pathogenic microorganisms.

Among isothermal nucleic acid amplification techniques, the LAMP assay typically requires at least 60 min to complete amplification and relies on a minimum of three primer pairs, which substantially increases the difficulty of primer design and optimization [36,37]. In contrast, RPA enables efficient nucleic acid amplification at a constant 39 °C without the need for thermal cycling instrumentation, thereby greatly simplifying operational procedures and diagnostic requirements [28]. Moreover, RPA is capable of accomplishing target amplification within 30 min, representing one of the fastest isothermal amplification strategies currently available. Nevertheless, despite its prominent advantages, RPA-based amplification occasionally generates non-specific amplicons, which may compromise the diagnostic accuracy and specificity of the assay [38].

The CRISPR/Cas12a system overcomes this drawback through its strict sequence-specific recognition guided by CrRNA [27]. Our closed-tube architecture—with the RPA reaction mixture at the tube bottom and the CRISPR detection system preloaded on the tube lid—minimizes aerosol contamination, a major source of false positivity in molecular diagnostics. Brief centrifugation after RPA mixes the two components, enabling seamless coupling of amplification and detection in a single closed vessel. This design is operationally simple and well-suited for field deployment.

The direct lysis and centrifugal nucleic acid extraction procedure reduced assay time and enhanced usability. This strategy has been previously documented and validated, and our results using clinical samples further support its direct applicability in molecular testing [32]. Performance evaluation demonstrated that the developed assay achieves analytical characteristics comparable to qPCR. The limit of detection (LoD) was 10 copies/reaction, which is 10–100 times more sensitive than qPCR and LAMP [34,37]. While the analytical sensitivity of the RPA-CRISPR/Cas12a method and qPCR was equivalent, a small number of clinical samples showed inconsistent results, with two positive samples (RPA-CRISPR/Cas12a) testing negative in qPCR. Such discrepancies have also been observed in our previous methodological evaluations [28,29,30,31]. It is well recognized that RPA exhibits higher tolerance to common PCR inhibitors and complex dirty clinical samples compared with qPCR [39]. In this study, we further evaluated the nucleic acid purity of all clinical samples, and the OD260/280 ratios of all samples were within the acceptable range of 1.8–2.0, indicating qualified nucleic acid purity without obvious contamination or impurity interference. Therefore, the inconsistent results of the two individual samples cannot be simply explained by nucleic acid purity or conventional PCR inhibitors. The exact underlying reason for this minor discrepancy is worthy of further exploration, and we currently consider it still unclear and it needs to be investigated in future work.

Several limitations of this study warrant consideration and future improvement. First, the current assay is a single-target detection system and cannot simultaneously identify multiple viruses common in clinical co-infections. Second, signal readout relies on a fluorescence detector, preventing naked-eye visualization in resource-limited settings without electricity. Third, the liquid reagents require cold-chain storage, which may hinder distribution in remote areas. Future work will focus on: (1) developing a multiplex RPA-CRISPR/Cas12a assay for simultaneous detection of CIAV and other major avian viruses; (2) integrating lateral flow dipsticks for visual readout without optical instruments; and (3) optimizing lyophilization technology to enable room-temperature storage and transportation of reagents.

In conclusion, the one-tube integrated RPA-CRISPR/Cas12a assay established in this study provides a rapid, sensitive, specific, and equipment-friendly approach for CIAV detection. Future work will further integrate the entire RPA-CRISPR/Cas12a detection system into a portable suitcase-based platform [40]. This integrated case will allow convenient on-site testing and rapid epidemiological screening directly at poultry farms without reliance on sophisticated laboratory facilities.

## Figures and Tables

**Table 1:** The sequences of the RPA primers and CrRNAs.

Name	Sequence (5′-3′)
F	ACGCTAAGATCTGCAACTGCGGACAATTCA
R	TGGGAGYAGTGGTAATCAAGCTTTCTTTTA
CrRNA1	GCTCGCTTACCCTGTACTCG
CrRNA2	AAGAATGTGCCGGACTTGAG
CrRNA3	GCTCGCTTACCCTGTACTCG

**Table 2:** Repeatability test.

DNA Copies	Inter-Repeat Test		Intra-Repeat Test	
	Fluorescence Average Value ± SD	CV	Fluorescence Average Value ± SD	CV
10^6^	3823.3 ± 216.0	5.7%	3796.7 ± 195.5	5.2%
10^4^	2607.7 ± 152.8	5.9%	2614.3 ± 189.8	7.3%
10^2^	1634.0 ± 137.3	8.4%	1647.33 ± 132.3	8.0%

## Data Availability

The data presented in this study are available on request from the corresponding author due to privacy protection of research participants.

## Supplementary Materials

The following supporting information can be downloaded at: https://www.mdpi.com/article/10.3390/vetsci13060529/s1.
