# Supplementary material for: Rapid Detection of Chicken Infectious Anemia Virus Using a One-Tube RPA-CRISPR/Cas12a System
Source: Vet Sci. 2026 May 29;13(6):529. doi: 10.3390/vetsci13060529 (PMC13308451; doi:10.3390/vetsci13060529)

Table.S1 the RPA primer sequences

Name	Sequence(5'-3')
F1	ATCCGGATTGGTATCGCTGGAATTACAATC
R1	TAGCAGGATCGCTTCTTCGAGGGAGGCTTG
F2	ACCATCAACGGTGTTCAGGCCACCAACAAG
R2	CACATTCTTGAAACCAGTGCTTTCTGAACT
F3	ACGCTAAGATCTGCAACTGCGGACAATTCA
R3	TGGGAGYAGTGGTAATCAAGCTTTCTTTTA

Figure.S1 Electrophoresis validation of the RPA primers

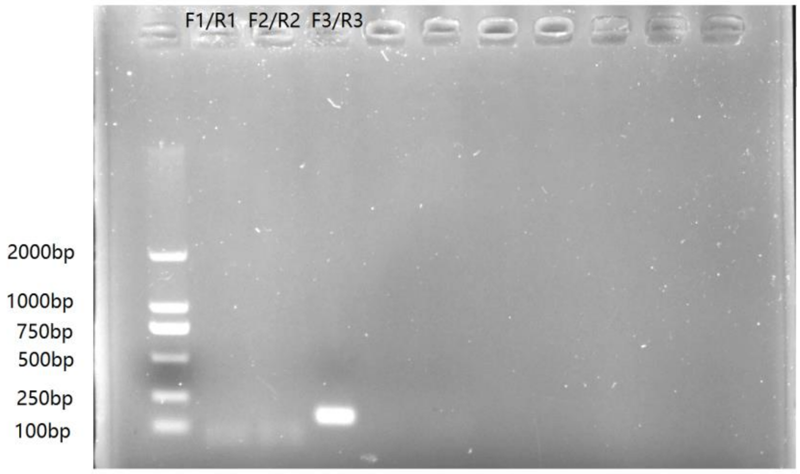

Figure.S2 the raw experimental results of the optimal CrRNA evaluation

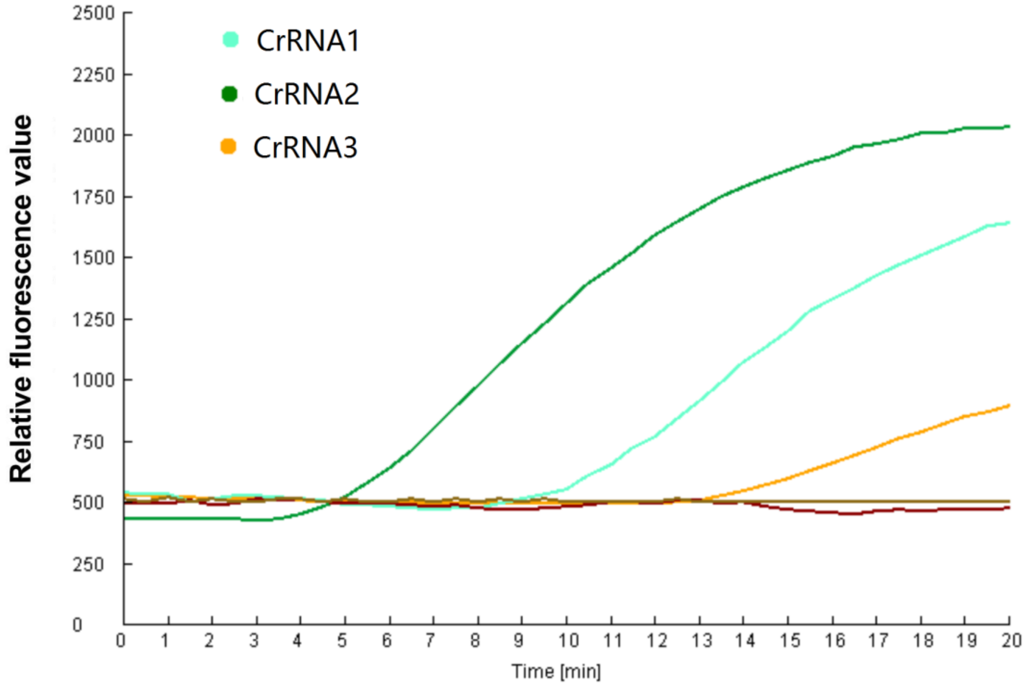

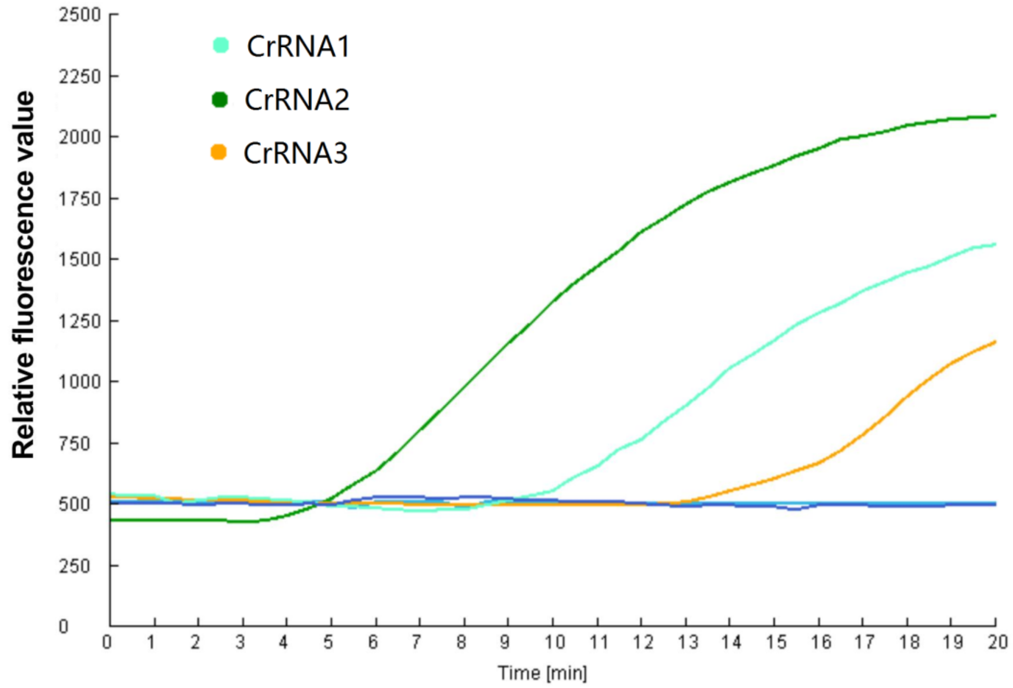

Figure.S2 Two independent experiments (10 replicates each, 10 copies/reaction)

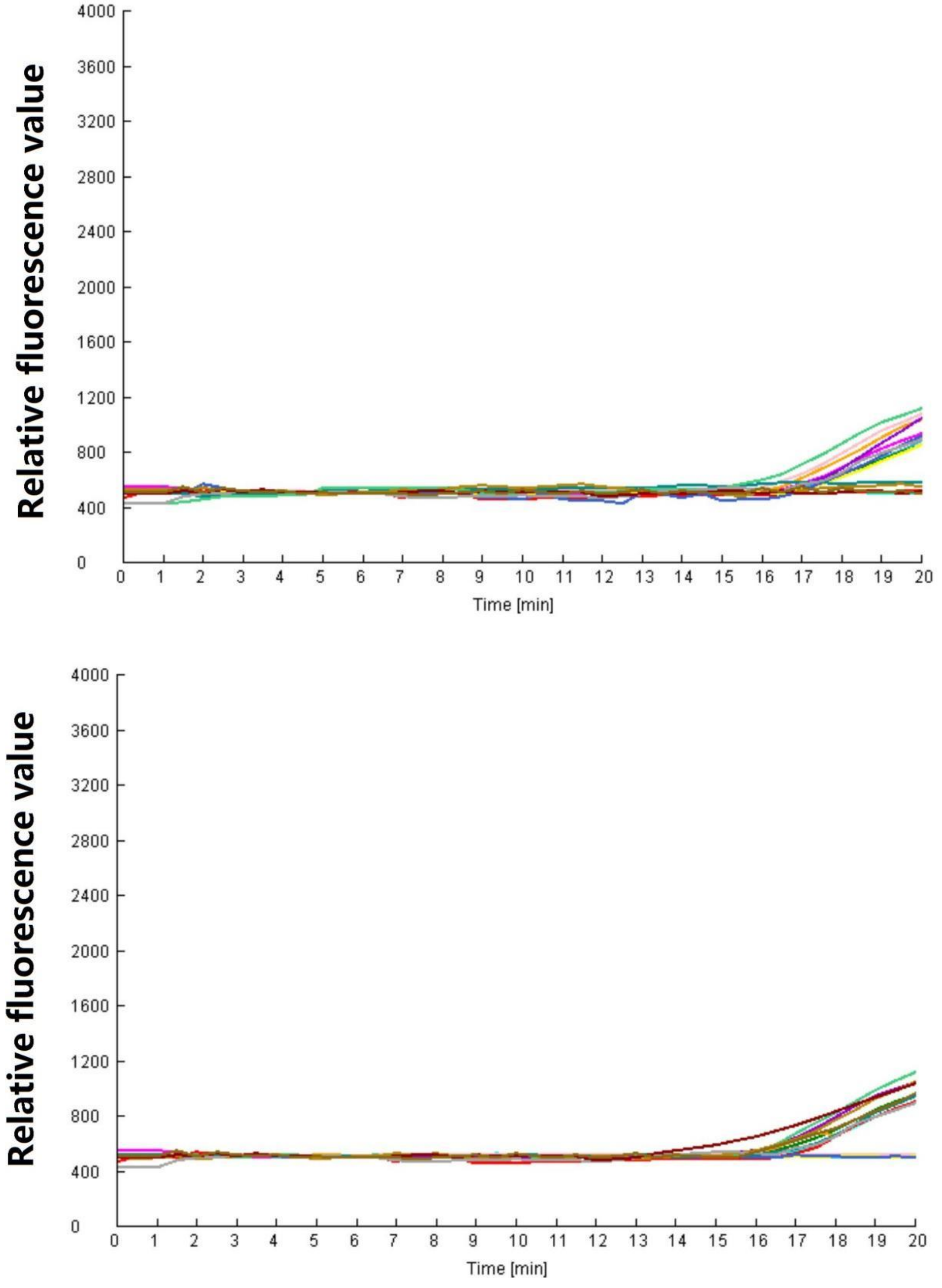

Figure.S3 Detection of clinical samples using the RPA-CRISPR/Cas12a assay

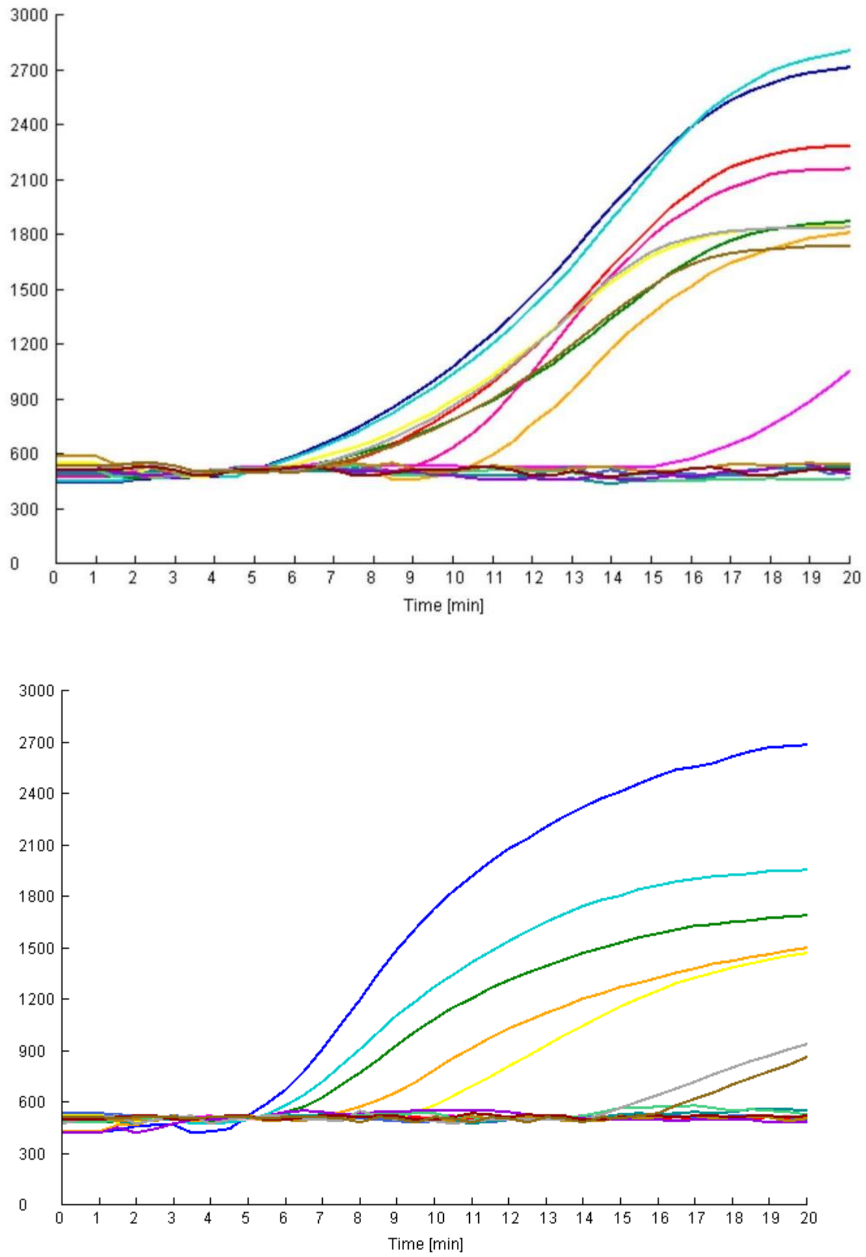

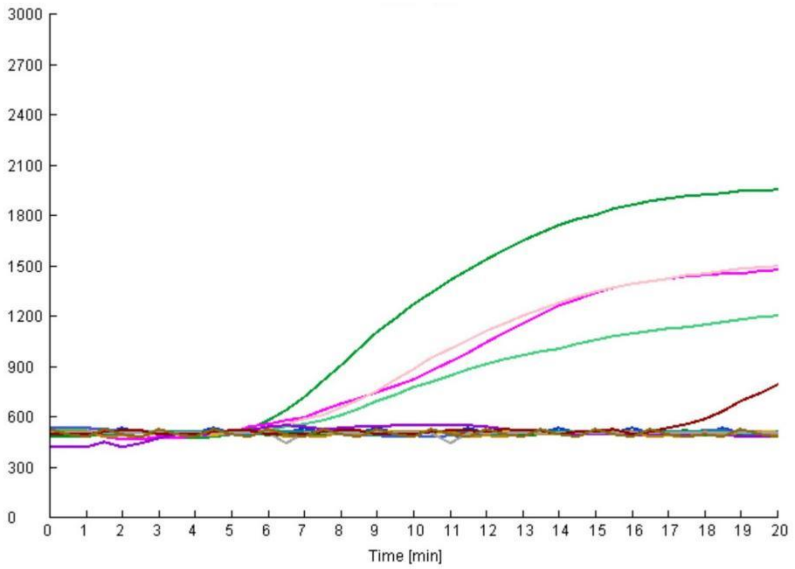
3000
2700
2400
2100
1800
1500
1200
900
600
300
0
0 1 2 3 4 5 6 7 8 9 10 11 12 13 14 15 16 17 18 19 20
Time [min]

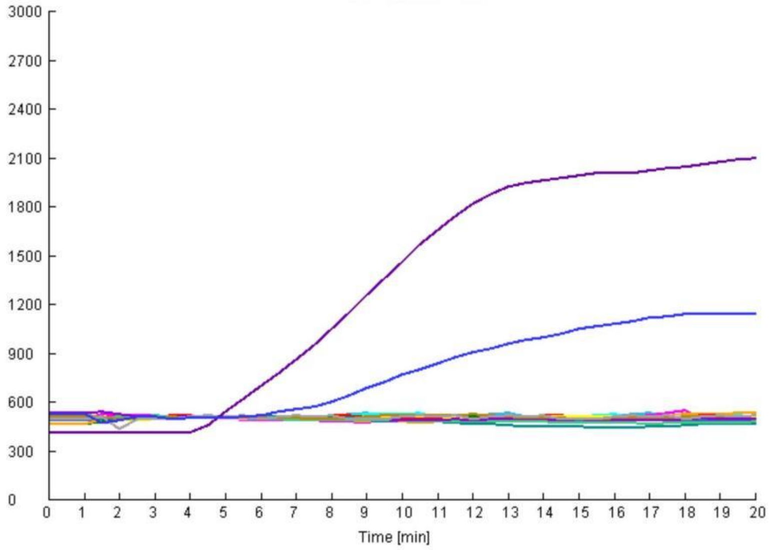
3000
2700
2400
2100
1800
1500
1200
900
600
300
0
0 1 2 3 4 5 6 7 8 9 10 11 12 13 14 15 16 17 18 19 20
Time [min]

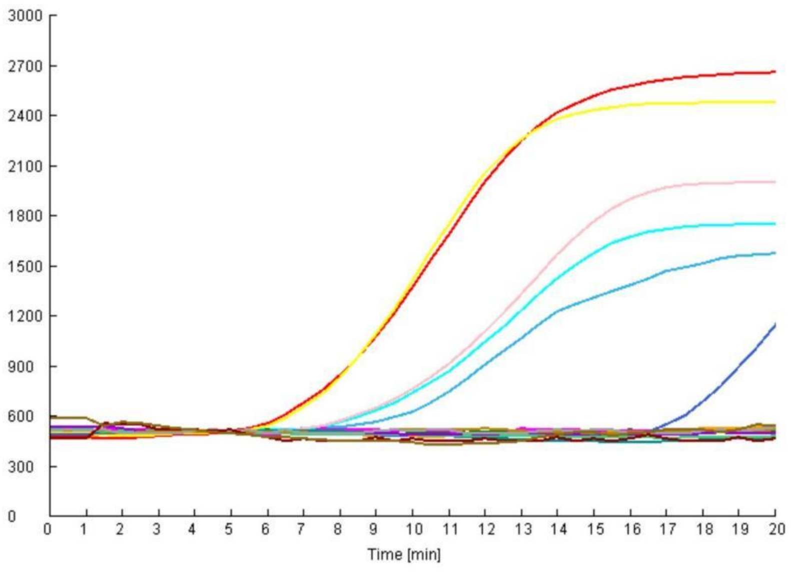
3000
2700
2400
2100
1800
1500
1200
900
600
300
0
0 1 2 3 4 5 6 7 8 9 10 11 12 13 14 15 16 17 18 19 20
Time [min]

Figure. S4 Detection of clinical samples using the qPCR assay

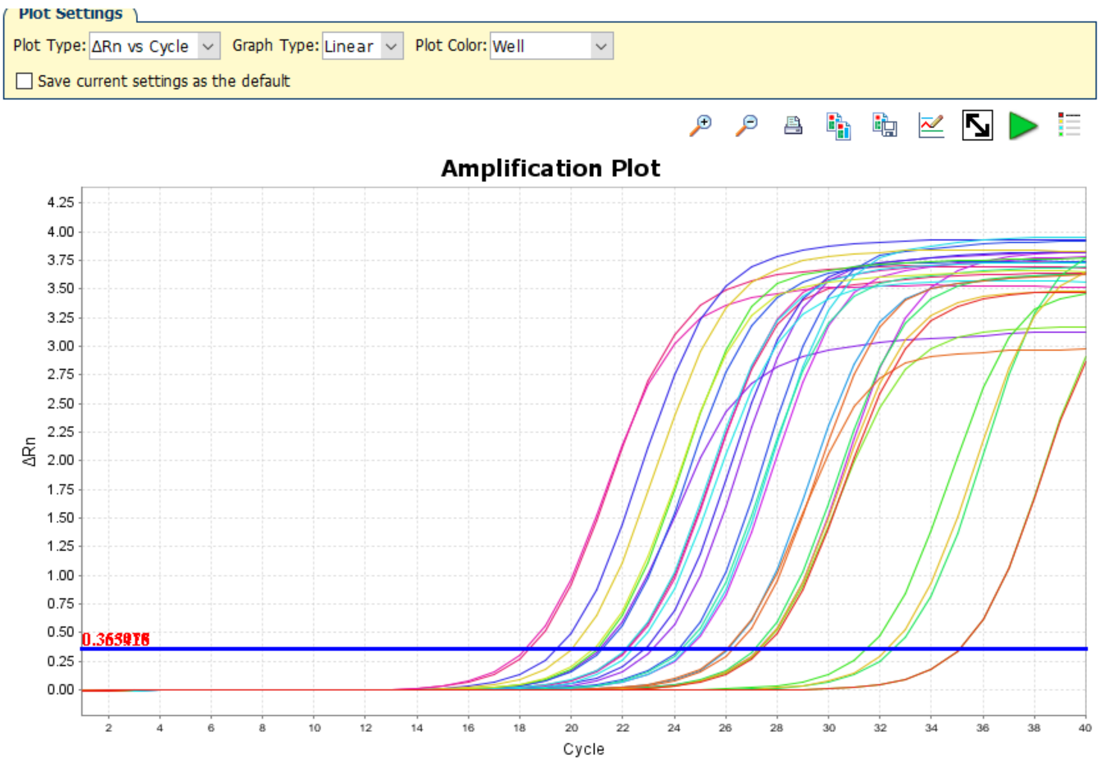

Supplement: Supplementary file 1 [file vetsci-13-00529-s001.zip › vetsci-4275199-supplementary.pdf]
